# Is Infusion of Subhypnotic Propofol as Effective as Dexamethasone in Prevention of Postoperative Nausea and Vomiting Related to Laparoscopic Cholecystectomy? A Randomized Controlled Trial

**DOI:** 10.1155/2015/349806

**Published:** 2015-01-28

**Authors:** Mine Celik, Aysenur Dostbil, Mehmet Aksoy, Ilker Ince, Ali Ahiskalioglu, Mehmet Comez, Ali Fuat Erdem

**Affiliations:** ^1^Department of Anesthesiology and Reanimation, Medical Faculty, Ataturk University, Palandoken, 25140 Erzurum, Turkey; ^2^Department of Anesthesiology and Reanimation, Erzurum Education Hospital, Palandoken, 25070 Erzurum, Turkey; ^3^Department of Anesthesiology and Reanimation, Sakarya Education Hospital, Korucuk Mah., 54290 Adapazari, Turkey

## Abstract

*Background*. Postoperative nausea and vomiting (PONV) is one of common complications in patients undergoing laparoscopic cholecystectomy (LC). Aim of this study was to compare the efficacy of subhypnotic (1 mg/kg/h) infusion of propofol with dexamethasone on PONV in patients undergoing LC. *Methods*. A total of 120 patients were included in this randomized, double-blind, placebo-controlled study. Patients were randomly assigned to 3 groups; patients of group dexamethasone (group D) were administrated 8 mg dexamethasone before induction of anesthesia, patients of group propofol (group P) were infused to subhypnotic (1 mg/kg/h) propofol during operation and patients of group control (group C) were applied infusion of 10% intralipid. The incidence of PONV and needs for rescue analgesic and antiemetic were recorded in the first 24 h postoperatively. *Results*. In the 0–24 h, the incidence of PONV was significantly lower in the group D and group P compared with the group C (37.5%, 40%, and 72.5%, resp.). There was no significant difference in the incidence of PONV and use of antiemetics and analgesic between group D and group P. *Conclusion*. We concluded that infusion of propofol 1 mg/kg/h is as effective as dexamethasone for the prevention of PONV during the first 24 hours after anesthesia in patients undergoing LC.

## 1. Introduction

Postoperative nausea and vomiting (PONV) is distressful common side effects following laparoscopic cholecystectomy (LC) [1, 2]. The reported incidence of PONV is 46–72% in patients undergoing LC if prophylactic antiemetic is not given [3, 4].

As an anesthetic agent, propofol is highly effective drug preventing PONV [5]; thus it has been used by a number of anesthesiologists [6]. It was demonstrated that continuous infusion of subhypnotic propofol prevents PONV in female patients receiving intravenous patient-controlled analgesia [7].

Glucocorticoids have analgesic, anti-inflammatory, immune-modulating, and antiemetic effects. But, their effect mechanisms are not fully clarified [8]. Dexamethasone is a glucocorticoid and has been used as an antiemetic drug in patients receiving chemotherapy for more than 25 years [9, 10]. Several prospective studies have shown that severity of PONV associated with LC is reduced by dexamethasone [11–13].

The primary aim of this prospective, randomized, double blind, placebo-controlled study was to evaluate the efficacy of dexamethasone and continuous infusion of subhypnotic propofol to prevent PONV in patients undergoing LC. Secondary aim of this study was to determine the rescue antiemetic and analgesic in the first 24 hours after LC.

## 2. Material and Methods

In this study, a total of 120 ASA I, II patients undergoing LC were included. The Institutional Review Board approved the study, and all 120 patients gave signed informed consent (Registration number: ACTRN: ACTRN12614000703606). Exclusion criteria were pregnancy, use of antiemetic drug 24 hours before LC, a history of nausea and vomiting in the previous operations, susceptibility to nausea and vomiting, menstruation, emergency operation, severe diabetes mellitus, and conversion from LC to laparotomy.

Noninvasive blood pressure, ECG, pulse oximetry, and capnometry were used for patient's monitoring during anesthesia. The patients were randomized using an equal number of blind envelopes and allocated to one of the three groups: dexamethasone group (group D), propofol group (group P), and control group (group C). Before one minute of anesthesia induction, while patients in group D received 8 mg of dexamethasone, group P and group C received isotonic saline solution in 2 mL syringe. The same anesthetic techniques were used for all patients. Anesthesia was induced by 5 mg/kg of thiopental sodium, 1 mcg/kg fentanyl through intravenous cannula. Intubation of the trachea was facilitated with 0.6 mg/kg of rocuronium and subsequent intraoperative neuromuscular blockade was maintained with it. Anesthesia was maintained with 1.0–2.5% sevoflurane air being given with 50% oxygen and 1 mcg/kg/h fentanyl half an hour. All patients were inserted a nasogastric tube after anesthesia induction to empty content and air of stomach. In group P, patients were given continuous propofol infusion at 1 mg/kg/h in during operation. In other two groups, suspension of 10% intralipid was infused in all patients. At end of surgery, neuromuscular blockade was reversed with neostigmine 0.05 mg/kg and 0.01 mg/kg atropine sulphate. For postoperative analgesia, after operation, 10 mL local anesthetic solution (equally mixed 2% lidocaine plus 0,5% bupivacaine) was applied to incision region and preemptive analgesia was performed via intravenous 1 g of paracetamol and 1.5 mg/kg of tramadol.

Awakening time was defined as patient's awakening (i.e., opening eyes at verbal command) and being orientated to the environment after discontinuation of the volatile agent.

All patients were observed for 24 hours by another anesthetist after surgery. These patients were informed about the scale of VAS (visual analog scale) and PONV by the anesthetist. The incidence of nausea, vomiting, and antiemetic requirement was recorded during three assessment periods, 0–6 h, 6–12 h, and 12–24 h after recovery from anesthesia using a four-point ordinal scale for PONV (0 = none, 1 = nausea, 2 = nausea with request for antiemetic, and 3 = vomiting). Rescue antiemetic (intravenously metoclopramide 10 mg) was allowed by the anesthetist according to needs of patients. Intramuscular diclofenac sodium (50 mg) as analgesic was medicated when patients experienced pain of VAS > 3.

Statistical analysis was performed using the program of SPSS20. One-way ANOVA was used to compare the differences of numeric data among the groups. Chi-squared test was used for categorical data. Level of significance was set at *P* < 0.05.

Before study, sample size was determined by power analysis, assuming that the total incidence of PONV in the placebo group would be 70%, with a 35% reduction in the incidence of PONV in the treatment group with alpha error being set at 0.05 and beta error at 0.2. According to power analysis, any group size of 31 patients was considered adequate. We decided to enroll 40 patients per group to allow dropout. The post-hoc test which was held during the statistical evaluation showed 31 patients for the propofol group and 35 patients for the dexamethasone group were needed.

## 3. Results

All 120 patients had completed their surgical procedures. There was no statistically significant difference among the 3 groups in terms of age, body weight and height, ASA classification, duration of anesthesia, surgery, and total fentanyl consumption (Table 1).

### 3.1. Primary Outcome

#### 3.1.1. Nausea and Vomiting

During 0–6 h, total incidence of PONV was 65% in the group C, 30% in the group P, and 30% in the group D. For 6–12 h, it was 52.5% in the group C, 25% in the group P, and 20% in group D. In 12–24 h period, it was 45% in the group C, 20% in the group P, and 10% in the group D. In group D PONV was significantly lower than in group C at 0–6 h (*P* = 0.007), 6–12 h (*P* = 0.06), and 12–24 h (*P* = 0.02). Also patients in group P had significantly less PONV than those of group C in the 0–6 h (*P* = 0.07), 6–12 h (*P* = 0.013), and 12–24 h (*P* = 0.039). There were no significant differences between the group D and group P with regard to PONV (Table 2).

### 3.2. Secondary Outcome

#### 3.2.1. Rescue Antiemetic

Four patients in group D, 4 patients in group P and 13 patients in group C were given antiemetic drug for 0–6 h. Patients in group D and group P had significantly less rescue antiemetic requirements than those of group C in this period (*P* = 0.01 for both). One patient in the group D, 3 patients in the group P, and 8 patients in the group C were in need of rescue antiemetic drug during 6–12 h. Patients in group D had significantly lower antiemetic drug requirement than those of group P (*P* = 0.01). There were no significant differences among the groups in 12–24 h in terms of antiemetic drug requirement (Table 2).

#### 3.2.2. Analgesic Requirements

In 0–24 h, 2 patients in the group D, 3 patients in the group P, and 8 patients in the group C were treated with diclofenac sodium 50 mg via intramuscularly and difference between group D and group C was significant (*P* = 0.04) (Table 3).

## 4. Discussion

Laparoscopic surgery has been associated with high incidence of PONV [3, 4]. PONV is an disagreeable, distressful, and fatiguing complication for patients undergoing LC. It might prolong recovery and discharge time therefore hospital costs increase [13]. In our study, PONV in dexamethasone group and propofol group was significantly reduced compared with control group. Therefore we found that infusion of propofol during the operation was as effective as dexamethasone to prevent PONV.

The analgesic effects of the glucocorticoids are mainly related to the inhibition of the phospholipase enzyme pathway. Additionally they also decrease in proinflammatory mediators such as interleukin-6 [14].

Dexamethasone use as an antiemetic agent in patients receiving cancer chemotherapy dates back to 1981 [9]. Although the mechanism of the antiemetic effect of dexamethasone is not fully understood it was suggested that dexamethasone may change in the permeability of the blood-brain barrier to serum proteins and inhibit endogenous opioid release and central prostaglandin synthesis [15, 16].

In one study, a single dose of glucocorticoid was given at different times during the perioperative period (perioperative period is defined as the time interval 12 hours before surgery until the end of surgery) in elective surgical procedures and it was found that glucocorticoid reduced postoperative pain and vomiting and nausea in all application times [17] We preferred to administer dexamethasone at 1 minute before anesthesia and found that this application of 8 mg dexamethasone reduced postoperative rescue analgesic requirements and PONV.

Borgeat et al. [18] demonstrated that propofol in subhypnotic doses (10 mg) possesses direct antiemetic properties in the context of minor elective surgery. Furthermore, the use of propofol for maintenance of anesthesia has a positive effect on PONV reduction [19]. Area postrema has the highest concentration of the 5 HT3 receptors in the brain. Possible stimulation of the 5 HT3 receptors in the area postrema with propofol may cause antiemetic effect. The authors found that the levels of serotonin were reduced in the area postrema and cerebrospinal fluid in propofol administered rats. Thus, antiemetic properties of propofol may be attributed to its weak serotonin antagonistic effect [20].

Song et al. [21] administered propofol 0.5 mg/kg intravenously at the end of a surgical procedure. They found that it is effective for preventing PONV in patients undergoing LC even at this dose. On the other hand, the small dose of propofol (0.5 mg/kg) administered at the end of surgery prolonged the times to awakening and orientation, but it did not delay the time to discharge from the postanesthesia care unit [21]. In another study, authors found that PONV was reduced significantly in the total intravenous anesthesia with propofol group compared to isoflurane-nitrous oxide anesthesia group [22].

Erdem et al. [23] used subhypnotic propofol infusion plus dexamethasone to prevent PONV in children undergoing tonsillectomy. In this study, the authors found that subhypnotic propofol infusion added to dexamethasone is more efficient than dexamethasone alone. Also, we demonstrated that propofol infused at a rate of 1 mg/kg/h for during of operation reduced incidence of PONV did not prolong awakening time.

Propofol used for the induction and maintenance of anesthesia effectively reduced early (0–6 h) PONV incidence in postoperative period [22]. In our study, early PONV incidence was similar in dexamethasone and propofol group. So we can suggest that propofol was as effective as dexamethasone for early PONV.

In group D and group C, we infused 10% intralipid as a placebo. Ostman et al. [24] demonstrated that intralipid have not antiemetic effect. So intralipid may be placebo for propofol, particularly in study of emesis.

Glucocorticoids have analgesic and antiemetic effects when administered perioperatively [25]. Data suggest that the pain-reducing effects of glucocorticoids can be secondary to a decrease in local edema [17]. Our study demonstrated that analgesic requirement in postoperative (0–24 hour) was less in patients given dexamethasone than in the control group. Limitations of this study: operation and anesthesia times were longer than other study [26]. While this situation was not to create a significant difference between the groups, incidence of the PONV may be increased.

## 5. Conclusion

We concluded that propofol 1 mg/kg/h is as effective as dexamethasone for the prevention of PONV during the first 24 hours after anesthesia in patients undergoing LC. Furthermore, dexamethasone effectively reduced the rescue analgesic requirement, while subhypnotic propofol infusion did not.

## Figures and Tables

**Table 1:** Demographic and operative characteristics of patients.

	Group D (*n* = 40)	Group P (*n* = 40)	Group C (*n* = 40)
Age (years)	49.6 ± 11.7	50.07 ± 12.1	49.9 ± 12.6
Weight (kg)	74.5 ± 13.3	74.2 ± 12.9	73.9 ± 13.5
Height (cm)	169 ± 9.7	168.1 ± 10.5	168.7 ± 10.1
Sex (M/F)	23/17	24/16	25/15
ASA (I/II)	27/13	29/11	28/12
Smokers (*n*)	11	10	12
Duration of surgery (min.)	78.5 ± 14.2	77 ± 13.9	80.2 ± 14.4
Duration of anesthesia (min.)	101.8 ± 9.5	99.9 ± 10.5	98.7 ± 11.6
Fentanly administered (*µ*g)	173.1 ± 55.2	169.3 ± 55.8	170.6 ± 53.9
Awakening time (min.)	5.9 ± 1.24	6.2 ± 1.21	6.1 ± 1.2

Values are *n* or mean ± standard deviation. ASA: American Society of Anesthesiologists classification.

**Table 2:** Incidence of nausea and vomiting during 24 h postoperatively.

	Group D (*n* = 40)	Group P (*n* = 40)	Group C (*n* = 40)
Scale of PONV 0–6 hours			
none	28 (70%)	27 (67.5%)	14 (35%)
Nausea	8 (20%)	6 (15%)	12 (30%)
Nausea with request for antiemetics	4 (1%)	2 (5%)	10
Vomiting	0	4 (10%)	4
Total PONV (*n*, %)	12 (30%)	12 (30%)	**26 (65%)** ^∗#^
Rescue antiemetic (*n*)	4	4	**13** ^∗#^

Scale of PONV 6–12 hours			
None	32 (80%)	29 (72.5%)	19
Nausea	8 (20%)	10 (25%)	13
Nausea with request for antiemetics	0	0	2
Vomiting	0	0	6
Total PONV (*n*, %)	8 (20%)	10 (25%)	**21 (52.5%)** ^∗#^
Rescue antiemetic (*n*)	1	3	**8** ^*^

Scale of PONV 12–24 hours			
None	36 (90%)	31 (77.5%)	22 (55%)
Nausea	4 (10%)	7 (17.5%)	16 (40%)
Nausea with request for antiemetics	0	1 (2.5%)	0
Vomiting	0	0	2 (5%)
Total PONV (*n*, %)	4 (10%)	8 (20%)	**18 (45%)** ^∗#^
Rescue antiemetic (*n*)	0	1	3

0–24 hours			
Total PONV (*n*, %)	**15 (37.5%)**	**16 (40%)**	**29 (72.5%)** ^∗#^

^*^Compared with group D *P* < 0.05, ^#^compared with group P *P* < 0.05.

**Table 3:** Analgesia (diclofenac sodium 50 mg) requirements.

	Group D (*n* = 40)	Group P (*n* = 40)	Group C (*n* = 40)
Diclofenac sodium requirements (patient numbers)	2 (5%)	3 (7.5%)	8 (20%)^*^

^*^ *P* < 0.05 compared with group D.
